# Turing Patterns with Cellular Computers

**DOI:** 10.1016/j.cels.2024.11.015

**Published:** 2024-12-18

**Authors:** Lewis Grozinger, Ángel Goñi-Moreno

**Affiliations:** 1 Systems Biology Department, Centro Nacional de Biotecnología (CNB), CSIC, Darwin 3, 28049 Madrid, Spain

## Abstract

Turing patterns are a key theoretical foundation for understanding organ development and organization. While they have been found to occur in natural systems, implementing new biological systems that form Turing patterns has remained challenging. To address this, Tica et al.^1^ used synthetic genetic networks to engineer living cellular computers that successfully generate Turing patterns within growing bacterial populations.

Over 70 years ago, Alan Turing developed a theory of morphogenesis which explained how complex spatial patterns could arise from spatially uniform initial states. He described remarkably simple systems of chemical reactions and diffusion laws that could generate all kinds of patterning seen in the natural world^2^, like in the pigmentation of pufferfish skin, or the asymmetrical structures that shape organ development. These kinds of systems are now called reaction-diffusion systems and their study shows how chemical reactions and diffusion together can generate complex, location-specific patterning in biological systems.

Although these Turing patterns have been well-studied in mathematics and computational systems for decades, progress in engineering physical implementations has been made only as recently as 2014^3^. And although reaction-diffusion systems in general have been engineered in living cells^4^, attempts at designing and building Turing patterns in living cells have been less successful. The main challenge has been that Turing pattern formation is typically very sensitive to the rate parameters that govern chemical reactions and diffusion in the system^5^. This makes it difficult to tune the parameters of engineered biological networks in order to realise pattern formation, even if the topology of the network is theoretically capable of producing patterns.

Now, Tica et al.^1^ have met this challenge, designing and constructing a genetic circuit in *Escherichia coli* cells that successfully generates concentric stripe patterns in growing populations. Their study has successfully merged careful mathematical, computational and experimental work to demonstrate convincingly the emergence of Turing patterns in a living synthetic biological system. They have also implemented control strategies to adjust genetic circuit parameters experimentally, allowing exploration of their system’s capacity to generate diverse patterns in response to parameter variation.

This success begins with previously published *in silico* work which surveyed genetic circuit topologies capable of producing Turing patterns^6^. This survey confirmed that the capability to produce Turing patterns is prevalent amongst genetic circuit topologies, but is extremely sensitive to rate parameter values, that is to say that Turing patterns are “common but not robust”^6^. Nevertheless, the work developed quantitative measures of robustness that were used to identify the most promising genetic circuit topologies for realisation, a crucial initial step upon which the present experimental work is built.

Tica et al.’s^1^ pattern generating circuit (Figure 1A) was selected based upon those findings. Key components of the circuit are two diffusible molecules and two reporter proteins. The diffusible molecules enable the emergence of Turing patterns by diffusing across cell membranes and interacting with the intracellular circuits of neighbouring cells. The genetic circuit expresses reporter proteins to display green or red fluorescence based on each cell’s internal state, allowing the researchers to visualize the pattern formation.

After constructing the circuit and inserting it into *E. coli* cells (Figure 1B), the populations exhibited various patterns dependent on experimental conditions. Conditions were adjusted in multiple ways to increase the diversity of patterns that emerged. This included controlling the intensity of circuit interactions by introducing specific chemical compounds (ATC and IPTG) in varying amounts to modulate circuit dynamics, as well as altering physical conditions such as agar thickness and the size of culture wells. As a result (Figure 1C) the authors were able to observe a variety of spatio-temporal patterns experimentally, such as stationary rings, propagating waves and oscillation, with differing brightness, size, and periodicity. Importantly, the authors used a computational model and simulations to predict or explain the effects of many of these variables on the experimental system and the emergence of the different patterns. This combination of theoretical and experimental work provides good evidence that Turing mechanisms are indeed responsible for the observed pattern formation.

Turing’s mathematical theory of morphogenesis was inspired by complex spatial patterns in Nature. Decades later, Tica et al.^1^ have now developed tools which open the door to further exploration of these Turing patterns in the context of engineered biological systems. On the theoretical side, stochasticity, delay and growth all have potential to push further in the direction of system robustness^7,8^. Experimentally, there is a richness of Turing patterns waiting to be targetted^5^. Opportunities and future directions abound.

Moreover, Tica et al.^1^ have demonstrated that they can use experimental conditions to control their pattern generating genetic circuit and produce diverse results as output. Such advances in synthetic biology and biocomputing are making it possible to engineer living cellular computers capable of entirely new, nature-inspired biocomputing functions^9^. The prospect of “programmable” spatio-temporal patterns in populations of living cells is especially exciting considering that such systems are distributed, multicellular and stochastic, all features of biology highlighted as key to the future success of biocomputing^10^.

## Figures and Tables

**Figure 1:**
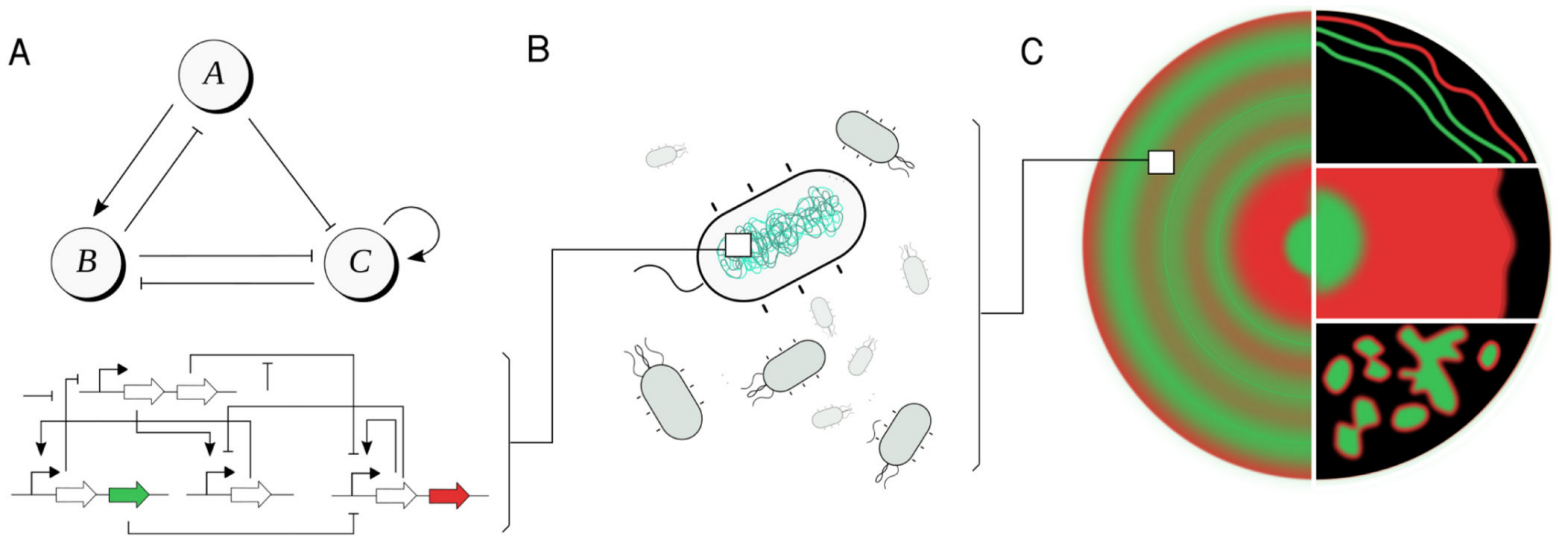
From gene circuit engineering to pattern control. **A**. Top: *In silico* analysis produced a promising functional three-node network design where nodes interact through either negative or positive regulation. Bottom: The corresponding genetic circuit has six genes including two fluorescent proteins, green and red, used to visualize the patterns in experimental results. **B**. Genetic circuits were implemented in *Escherichia coli* cells, with each cell in the population carrying the same genetic program. **C**. Radial patterns (left) were successfully and reproducibly observed in cell populations. Various other patterns (right) were obtained through experiments and/or simulations which explored a broad range of experimental conditions.
